# US medical specialty global health training and the global burden of disease

**DOI:** 10.7189/jogh.03.020406

**Published:** 2013-12

**Authors:** Vanessa B. Kerry, Rochelle P. Walensky, Alexander C. Tsai, Regan W. Bergmark, Brian A. Bergmark, Chaturia Rouse, David R. Bangsberg

**Affiliations:** 1 Center for Global Health, Massachusetts General Hospital, Boston, Mass, USA; 2 Department of Global Health and Social Medicine, Harvard Medical School, Boston, Mass, USA; 3 Division of Pulmonary and Critical Care, Department of Medicine, Massachusetts General Hospital, Boston, Mass, USA; 4 Division of General Medicine, Department of Medicine, Massachusetts General Hospital, Boston, Mass, USA; 5 Division of Infectious Disease, Department of Medicine, Massachusetts General Hospital, Mass, USA; 6 Division of Infectious Disease, Department of Medicine, Brigham and Women’s Hospital, Boston, Mass, USA; 7 Center for AIDS Research, Harvard University, Cambridge, Mass, USA; 8 Chester M. Pierce, MD Division of Global Psychiatry, Department of Psychiatry, Massachusetts General Hospital, Boston, Mass, USA; 9 Department of Otolaryngology – Head and Neck Surgery, Massachusetts Eye and Ear Infirmary, Boston, Mass, USA; 10 Department of Otology and Laryngology, Harvard Medical School, Boston, Mass, USA; 11 Department of Medicine, Brigham and Women’s Hospital, Boston, Mass, USA; 12 Boston University, Boston, Mass, USA; 13 Mbarara University of Science and Technology, Mbarara, Uganda

## Abstract

**Background:**

Rapid growth in global health activity among US medical specialty education programs has lead to heterogeneity in types of activities and global health training models. The breadth and scope of this activity is not well chronicled.

**Methods:**

Using a standardized search protocol, we examined the characteristics of US medical residency global health programs by number of programs, clinical specialty, nature of activity (elective, research, extended curriculum based field training), and geographic location across seven different clinical medical residency education specialties. We tabulated programmatic activity by clinical discipline, region and country. We calculated the Spearman's rank correlation coefficient to estimate the association between programmatic activity and country–level disease burden.

**Results:**

Of the 1856 programs assessed between January and June 2011, there were 380 global health residency training programs (20%) working in 141 countries. 529 individual programmatic activities (elective–based rotations, research programs, extended curriculum–based field training, or other) occurred at 1337 specific sites. The majority of the activities consisted of elective–based rotations. At the country level, disease burden had a statistically significant association with programmatic activity (Spearman's ρ = 0.17) but only explained 3% of the total variation between countries.

**Conclusions:**

There were a substantial number of US medical specialty global health programs, but a relative paucity of surgical and mental health programs. Elective–based programs were more common than programs that offer longitudinal experiences. Despite heterogeneity, there was a small but statistically significant association between program location and the global burden of disease. Areas for further study include the degree to which US–based programs develop partnerships with their program sites, the significance of this activity for training, and number and breadth of programs in medical specialty global health education in other countries around the world.

United States (US) – based academic global health programs have more than quadrupled in number between 2003 and 2009 [1]. These programs are characterized by research, clinical practice, or education that aims to improve understanding of the root causes of disease and better care delivery models to vulnerable populations across geographic borders. Global health education often includes material about the social, economic, environmental, historical, and political determinants of health, with a goal of health equity for all [2].

The rapid expansion of US global health programs has been multifactorial. Medical students and graduates are increasingly seeking to matriculate to residency and fellowship programs at academic centers which offer opportunities in global health. In one study, 92% of US surgical residents surveyed expressed interest in an international elective, and 82% noted they would prioritize a global health elective over any other [3]. Similarly, 90% of family medicine applicants considered global health as an essential program component [4]. From a program perspective, US global health training activities have been supported by increases in federal and foundation funding, and also figured prominently in recruiting of top applicants. In recent studies, both emergency and family medicine residency applicants ranked programs with global health rotations over those that did not offer global health rotations [4,5].

This rapid growth has been largely uncoordinated between training institutions and, therefore, is at risk of not necessarily aligning training focus to optimize the experience with the global burden of disease [6]. To date, there has been little consensus, guidelines or bench marks regarding what comprises core competencies in global health education and training [7,8].

While related work has examined the scope of US post–graduate medical specialty training in global health [9,10] and the relationship between national and international funding priorities and disease burden [11-13], to date there has been no comprehensive review of the variation in global health education and programmatic activity with respect to structure, disease focus, and geographic distribution. One method of obtaining such information would be to directly survey program directors as has been done in specific specialties. However, such surveys are challenged by very low response rates ranging from 25–59% [10,14,15]. Surveys of program websites have been performed previously [16] but none have specifically focused on global health training. To help address these gaps in the literature, we systematically collected characteristics of global health programs in US academic medical specialty training programs (residency) from web based program descriptions – a more sensitive method compared to survey – to catalogue existing programs as a first step towards understanding the breadth of global health education in different specialties; our secondary aim was to understand their distribution relative to the global burden of disease. Our goal was to characterize existing programs with respect to geography, specialty, and programmatic activity and to compare how these characteristics map to the global burden of disease.

## METHODS

We systematically collected information available on residency program websites of seven major US graduate medical education specialties. Our search aimed to identify the presence of global health–focused training type of activity, and geographic location of these global health programs.

### Program identification

Clinical residency training programs including internal medicine, pediatrics, general surgery, obstetrics and gynecology, mental health, emergency medicine and family medicine were compiled from the official Accredited Council for Graduate Medical Education (ACGME) website [17]. These programs are seven of the largest residency specialties in number of trainees and represented over 50% the total residents (N = 115 546) in the United States [17] in residency programs during academic year 2010–2011. Subspecialties, fellowships and joint clinical programs (eg, medicine/pediatrics) were excluded.

### Search protocol

From January – June 2011, the following terms were entered (without punctuation) as separate queries using Google as a web search engine: “[Insert ACGME–listed program’s name] global health [Insert Clinical Discipline] residency.” The web page results were reviewed by one of several trained data abstractors (CR, RB, BB, VBK) for the first 20 search results [18,19]. Search protocols included an examination of web pages for all links that included any of the following keywords: “global health,” “international health,” “enrichment,” “rural,” “research,” “vulnerable populations” or “health inequity.” Data were also abstracted from every webpage linking the ACGME–accredited program to any of the following: “residency program,” “clinical training,” “research,” “rotation” or “curriculum” related to global or international health. If the first 20 Google queries and the program website did not mention a program related to global health, then that program was coded as not having one. Programs listing only general and unspecified terms for electives were also excluded. The complete URL of each queried webpage was recorded. Fifteen percent of web searches were queried by a second reviewer (VBK) to confirm reproducibility of the information obtained. The primary function of this confirmatory check was to serve as a quality control mechanism, so agreement statistics were not calculated. Google searches were performed after clearing all browser cookies and signing out of Google accounts, so as to minimize the personalization of results to a specific user.

### Program characteristic definitions

Global health training programs were evaluated for three types of programmatic activity: “elective–based rotations,” “research programs” or “extended curriculum–based field training.” Elective–based rotations were defined as clinical or educational activities of less than six weeks duration. Research programs required some component of data collection or human subjects approval. Extended curriculum–based field training experiences were defined as engagements greater than six weeks and/or including a designated course of study concentrating on pertinent principles in global health. If an activity did not fit the previous three classifications or could otherwise not be characterized, it was listed as “other.” Sites of programmatic activity were designated as country(ies) where any of the three above the programmatic activities occurred. To categorize these programmatic sites, we used the designated World Health Organization (WHO) list of countries and regions [20], specifying six regions: Africa, Americas, Eastern Mediterranean, Europe, Western Pacific and Southeast Asia. The study was completed before South Sudan’s independence. The United States was excluded in final results as federal reimbursement of medical education is determined in part by activities in resource limited areas in the US; all US programs would meet the outlined criteria [21,22].

### Analysis

The data were tabulated and summary descriptive statistics were used to compare program characteristics by region. Data from the 2004 WHO Global Burden of Disease assessment [20] were used for comparison to programmatic density by discipline, region and by country. To estimate the association between programmatic activity and disease burden, we calculated the Spearman’s rank correlation coefficient between the two variables. We fit an ordinary least squares regression model to the data with the number of programs as the dependent variable and the burden of disease (per 100 000 DALYs) as the exposure of interest, with a cluster–correlated robust estimate of variance to account for potential clustering of observations within countries [23-25]. Disease–burden elasticity of program existence (ie, percent change in existing programs in relation to a percent change in disease burden) was evaluated at the means. In a sensitivity analysis, we constrained the intercept to be zero so as to mimic a process in which countries with no disease burden had no programmatic activity [12]. Statistical analyses were conducted using the Stata/MP software package (version 12.0, StataCorp LP, College Station, Tex., USA). Density of programs per country was categorized into seven defined cohorts (indicated in legend); these cohorts were then translated into a color–coded map using StatPlanet software by StatSilk (version 3.0, StatPlanet, Melbourne, Australia).

## RESULTS

A total of 1856 ACGME residency programs were identified in internal medicine, pediatrics, obstetrics and gynecology (OB/GYN), general surgery, emergency medicine, family medicine, and psychiatry (**Table 1**). Three hundred–eighty (20%) of the total residency programs evaluated had documentation of global health training programs, with a total of 529 programmatic activities. The majority of programmatic activities consisted of elective–based rotations (292 [55%]), followed far behind by research programs (122 [23%]) and then extended curriculum–based field training (84 [16%]); thirty–one program activities (6%) could not be categorized because the type of activity was not explicitly described. When disaggregated by discipline, all seven disciplines had more elective–based rotations relative to any other activity type. The greatest number of elective–based programs was in family medicine (66 [62%]), whereas the greatest number of research programs was in emergency medicine (31 [30%]). Psychiatry had the lowest number of programmatic activities of any specialty, followed closely by general surgery. Within specialties, family medicine (17 [18%]), pediatrics (19 [19%]), and internal medicine (21 [20%]) all had a high–frequency of extended curriculum–based field experiences. All seven specialties demonstrated all three types of programmatic activities.

**Table 1:** Number of global health training programs and programmatic activities per clinical specialty

Specialty	Number of total ACGME residency programs per specialty	Number of residencies with global health training programs (% total in specialty)	Number of residencies with global health training programs (% total of 380 global health programs)	Total number of programmatic activities by specialty (mean programmatic activities per global health training programs in each specialty)*	Number (%) of programs with elective–based activities	Number (%) of programs with research programs	Number (%) of programs with extended curriculum–based field training	Number (%) of programs with other activities
Internal medicine	380	75 (20)	75 (20)	97 (1.3)	51 (53)	18 (19)	17 (18)	11 (10)
Pediatrics	198	65 (33)	65 (17)	101 (1.6)	59 (58)	20 (20)	19 (19)	3 (3)
OB/GYN	243	41 (17)	41 (11)	69 (1.7)	33 (49)	18 (26)	8 (11)	10 (14)
General surgery	246	21 (9)	21 (6)	33 (1.6)	14 (43)	12 (36)	5 (15)	2 (6)
Emergency medicine	155	64 (41)	64 (16)	105 (1.6)	59 (56)	31 (30)	8 (7)	7 (7)
Family medicine	451	97 (22)	97(26)	107 (1.1)	66 (62)	15 (14)	21 (20)	5 (5)
Psychiatry	183	17 (9)	17 (4)	28 (1.6)	10 (36)	8 (29)	6 (21)	4 (14)
**Total**	**1856**	**380 (20)**	**380 (100)**	**529 (1.4)**	**292 (55)**	**122 (23)**	**84 (16)**	**31 (6)**

ACGME – Accredited Council for Graduate Medical Education, OB/GYN – Obstetrics and gynecology

*The ratio represents the average number of programmatic activities per global health residency training program in a given specialty. A ratio of 1.3 means that, in internal medicine for example, 97 programmatic specialties over 75 global health training programs results in an average of 1.3 programmatic activities per program in that specialty.

**Figure 1:**
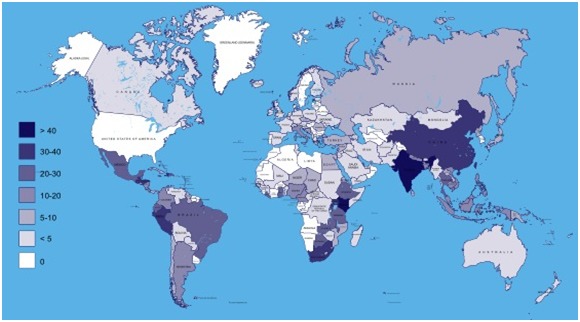
Density of programs by country. The legend on the left refers to the number of countries indicated by color. Each color corresponds to a set range of programmatic activities. Seventy–nine countries have fewer than 5 programmatic activities, 42 countries have only one program in the country and 53 have no reported activity. Websites offered insufficient detail to reliably discern the degree of bilateral exchange between programmatic activities. All of this programmatic activity was assumed to be based in partner country sites. The United States was excluded.

Geographic location by country was available fully or in–part for 223 (59%) of the total 380 global health training programs identified which demonstrated programmatic activity in 141 countries in the world. Thirty–nine global health training programs referenced having both programmatic activity in specific countries as well as programmatic activity that was not assigned a specific country. One hundred and fifty–seven global health training programs did not specify the countries in which they were working. The 529 individual programmatic activities occurred at 1337 specific sites (**Table 2**). Africa had the greatest number of programmatic activities (384 [29%]) overall and for all residency disciplines except emergency medicine and family medicine. The Americas had an almost identical number of total programmatic activities (369 [28%]). The Western Pacific (128 [9%]) and Southeast Asia (124 [9%]), the regions with the third and fourth highest density of activities, had less than half of either the Americas or Africa. Evaluated by the number of programmatic activities in each specific country, Kenya had the highest number with 60 different program activities followed by India (n = 50) and Haiti (n = 38) (**Figure 1**). Importantly, websites offered insufficient detail to reliably discern the degree of bilateral exchange between programmatic activities. All of this programmatic activity was assumed to be based in partner country sites.

**Table 2:** Programmatic activity by World Health Organization (WHO) region and clinical specialty*

Region	Total number of sites with programmatic activities (% of total 1337 sites)	Number of sites with programmatic activity by specialty (% of total per clinical discipline)						
		**Internal medicine**	**Pediatrics**	**OB/GYN**	**General surgery**	**Emergency medicine**	**Family medicine**	**Psychiatry**
Africa	384 (29)	123 (3)	84 (35)	53(34)	18 (30)	41 (20)	36 (20)	29 (30)
Americas	369 (28)	102 (26)	78 (32)	36 (23)	12 (20)	48 (23)	72 (39)	21 (22)
Western Pacific	128 (9)	41 (10)	20 (8)	14 (9)	9 (15)	24 (12)	11 (6)	9 (9)
Europe	95 (7)	33 (8)	15 (6)	9 (6)	3 (5)	15 (7)	8 (4)	12 (13)
Eastern Mediterranean	41 (3)	14 (3)	7 (3)	2 (1)	0 (0)	11 (5)	5 (3)	2 (2)
Southeast Asia	124 (9)	36 (9)	24 (10)	14 (9)	4 (7)	23 (11)	10 (5)	13 (13)
**Sub–total specified sites**	**1141 (85)**	**349**	**228**	**128**	**46**	**162**	**142**	**86**
Unspecified sites	196 (15)	46 (12)	13 (5)	26 (17)	14 (23)	44 (21)	42 (23)	11 (11)
**Total**	**1337**	395	241	154	60	206	184	97

OB/GYN = Obstetrics and gynecology

*The total number of programmatic activities was mapped according to WHO region. One hundred and ninety–six programmatic activities (15%) could not be mapped to a specific country.

**Figure 2:**
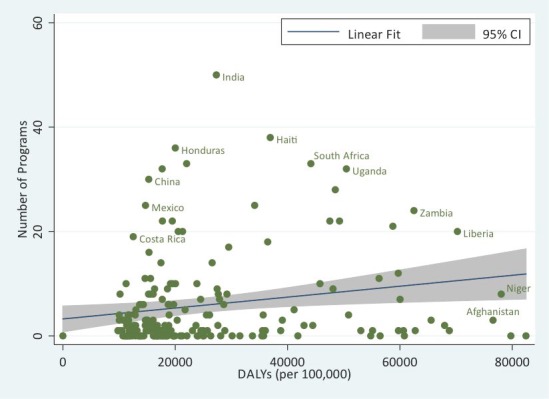
Intensity of programmatic activity by country–level burden of disease. Points above the fitted line represent countries that have a greater number of programs than predicted by our regression model, whereas points below the line represent countries that have fewer programs than predicted.

At the country level (n = 193), there was a statistically significant correlation between burden of disease and programmatic activity (Spearman’s ρ = 0.17; 95% CI, 0.03–0.31). Fitting a linear regression model, only a small proportion of the variance in programmatic activity could be explained (adjusted R^2^ = 0.03) (**Figure 2**). Each 10 000 Disability Adjusted Life Year (DALY) increment (indicating an increase in disease burden) per 100 000 persons in that country was associated with the existence of approximately one additional residency program (b = 0.0001; 95% CI, 0.00002–0.0002). Expressed differently, a two–fold increase in DALYs per 100 000 was associated with a 41% (95% CI, 11.6–71.1) increase in the number of residency program activities within a country. Several countries had a far greater intensity of program activity than would be predicted on the basis of disease burden alone, most notably India, Haiti, and Honduras. In the sensitivity analysis, a regression model with the intercept constrained at zero yielded a similar estimate.

## DISCUSSION

Our analysis found 20% of assessed US residency programs have global health programmatic activities and revealed a statistically significant correlation between country–level disease burden and the density of current US residency global health programs; this suggests that US residency program leadership feel there is a benefit from learning in a global setting and are directing resources to global health education. While the association between number of programs and disease burden for each country is statistically significant, there is substantial heterogeneity.

Global health programs span a broad range of clinical disciplines. They are more common, though, in general medical specialties such as internal medicine, emergency medicine, family medicine and pediatrics than more technically focused specialties such as obstetrics and gynecology or general surgery. The technical and service requirements of surgical training, including obstetrics and gynecology, may explain the paucity of programs in these fields. Surgery generally involves a complex care model requiring operating rooms, equipment, sterilization methods and anesthesia, many of which are absent or lacking in these global settings. Additionally, many surgical diseases have not been viewed traditionally as a public health problem and thus effort and funds have not been allocated as frequently as in other specialties [26]. Finally, there may be concern in domestic programs that electives abroad interrupt essential skill building.

We found few global health psychiatry programs relative to the global burden of psychiatric disease.

In resource–limited settings, neuropsychiatric disorders and suicides are a major cause of morbidity and mortality [27,28]. Compared to surgery and obstetrics, the paucity of global health psychiatry programs is less likely to be explained by technical and/or service requirements. However, the clinical practice of psychiatry relies more on cultural and language familiarity than other specialties. Accordingly, issues related to culture and language may pose a greater barrier to establishing psychiatry global health programs [29,30].

More than half of global health programmatic activity is rotation or elective–based and reflects a myriad of activities. The impact of “visitors” during elective rotations can be either positive, negative or somewhere in between. For example, elective rotations can occur at a site of long–term partnership with concrete supervision, goals and curricula and where they participate in the goals and mission of the site. Alternatively, residents may serve as “medical tourists” without integration into local systems. These latter programs can compromise both the resident’s experience and the functioning of the recipient site. Brief elective rotations have demonstrated improved clinical skills, increased cultural sensitivity, better public health awareness, greater appreciation of resource utilization and a more in depth understanding of the challenges of delivering care in resource–poor settings among US–based rotators [7,31,32]. The benefits for host countries of these brief stints for trainees are poorly characterized. Ultimately, the investment and dividends of each type of programmatic activity, whether elective–based, research or extended curriculum–based field training are broad. Each will have different impact on trainees from US institutions and the partner sites depending on level of funding, depth or partnership and priority setting. More research will be needed to characterize the potential benefits (or harms) of the programmatic activities.

With the constraint that educational programs require time to develop, we correlated the global health programmatic activity among residency programs in 2011 with the most recently reported global burden of disease data published in 2008. Our analysis revealed a statistically significant correlation between country–level disease burden and the density of current residency global health programs, suggesting that residency programs are directing resources to countries of greater need. However, the magnitude of association is small, and the policy relevance of this association is unclear. In the US national studies by Gillum et al. [12] and Gross et al. [13], 33–39% of the variance in National Institute of Health (NIH) funding could be explained by category–specific disease burden alone, whereas in our model only three percent of the variance in programmatic activity could be explained by country–level disease burden. Despite the statistical significance, the order–of–magnitude difference, however, is likely due to ad hoc rather than actively coordinated establishment of programs informed by priority–setting exercises such as those which have been performed for global child and mental health research [33-36]. As programs become more numerous and sophisticated, such studies could be convened by residency leadership bodies such as the American Association of Directors of Psychiatry Residency Training or the Association of Program Directors in Surgery. Long overdue, these studies could potentially provide valuable and systematic guidance to residency programs seeking to establish new training sites in order to maximize the collective impact on the global burden of disease [37].

While our study is able to characterize the distribution of global health programs by clinical discipline, the global burden of disease is not as easily partitioned. Many diseases may have multi–disciplinary care models such as malignancy, infectious diseases or trauma, or a clinical discipline such as family medicine might address multiple causes of morbidity and mortality. For example, conditions treatable by surgical intervention represent an estimated 11% of the global burden of disease [38-40] whereas according to our study, 9% of surgery residencies have global health training. Psychiatry represents 13% of the global burden of disease and one–third of years lost due to disability [41] but only 4% of global health residency training programs specifically address mental health. Future program growth should prioritize these disparities.

Our study was limited to US programs in medical specialty education and reflects a growing interest in global health engagement in the US. We believe this reflects a global trend based on known partnerships between institutions in resource limited countries and non–US institutions. For example, Bristol University in the United Kingdom partners with Mbarara University of Science and Technology in Uganda or the University of Naples in Italy has an exchange with Gulu University also in Uganda [42,43]. However, a direct comparison is difficult to make. Many international teaching and training partnerships remain scarcely recorded in the literature making it difficult to understand international trends in global health training programs. This phenomenon is especially notable among medical specialty education, or residency, programs. While there are publications on medical school global health education from North America, Europe, South America and the Pacific [9,44-49], literature for non–US residency education programs is scarce. Of note, a rare article on graduate medical education from Australia, reports that despite significant interest among trainees, global health education is not well developed [50].

There are several additional limitations to our findings. The internet–based protocol does not allow assessment of the degree of bilateral exchange, the depth of partnerships or opportunities for capacity building and education of partner trainees. It was not designed to capture the nature of programmatic activity in each country, the location of activity within each country, nor the details regarding sub–specialty (eg, infectious disease, cardiovascular disease or intensive care). Importantly, though not within the scope of this study, a deeper analysis of activities within countries would add to our findings. South Africa and India, for example, have very disparate burden of disease within the country. Understanding the location of activities for each program within certain countries would continue to refine the response to burden of disease.

The web based search protocol is limited in its sensitivity and will not capture global health activity of residencies which is not posted on their website [15]; activity may be informal or formal and not described at all or it possible that global health activities may be masked by generic terms such as “electives.” While a systematic survey of residency directors or administrators could potentially provide more in depth and current description of global health activities, previous efforts at surveys have yielded inconsistent and poor response rates, which may introduce additional biases [10,14,15]. We recognize that some institutions may create institution–wide, or cross–campus, initiatives to organize global health programmatic activities that may make it more likely for a specific residency program to establish a global health program once another residency program within the same institution has already established a global health program at a given site. However, we expect that such umbrella initiatives are rare relative to the overall degree of activity. It is also possible that these activities may not have been captured in our search results. For example, our estimates of surgical programs with global health training fall short of those captured in a 2009 study by Javaraman et al [14]. Finally, only seven residencies were evaluated out of approximately 30 clinical residency disciplines listed by the ACGME in 2011 [17]; while a defined sample, this large subset, reflects over 50% of all residents trained in the US in 2011. Because of the dynamic nature of programs or websites, updates may have been made since our initial data collection that were not included in this review. However, we believe this study provides an important overview and understanding of the trends in US medical specialty education and the global burden of disease.

Further evaluation will need to be conducted to better understand any additional granularity by specialty and/or subspecialty, as well as the depth of partnership between a US academic program and partner site and the amount of knowledge transfer. Equally, further evaluation would help elucidate the challenges to developing programs and international partnerships which may include cultural and language barriers, financial constraints, differing priorities between partner institutions or unsustainability. It will be important to better characterize the type of clinical education and investigation in each location.

## CONCLUSION

Characterizing global health education among medical specialties in the US is the first step to standardizing global health training at this level in order to improve the experience for our trainees and to determine the extent to which US global health education reflects and addresses the global burden of disease. Identifying gaps in today’s global health education will guide global health training to reduce the morbidity and mortality caused by the diverse etiologies of global burden of disease. The impact and benefits of these programs on trainees and vulnerable populations will need to be better assessed to balance the distribution of programs with respect to geography and disease burden and to better understand how to shape global health programs in medical specialty education.
